# The interRAI CHESS scale is comparable to the palliative performance scale in predicting 90-day mortality in a palliative home care population

**DOI:** 10.1186/s12904-022-01059-3

**Published:** 2022-10-06

**Authors:** Nicole Williams, Kirsten Hermans, Joachim Cohen, Anja Declercq, Ahmed Jakda, James Downar, Dawn M. Guthrie, John P. Hirdes

**Affiliations:** 1 Department of Kinesiology and Physical Education, Wilfrid Laurier University, 75 University Ave W, Waterloo, Canada; 2 LUCAS – Center for Care Research and Consultancy, KU Leuven, Minderbroedersstraat 8 box, 5310, 3000 Leuven, Belgium; 3 End-of-life Care Research Group, University of Brussels (VUB) and Ghent University (UGent), Laarbeeklaan 103, 1090 Brussels, Belgium; 4 Department of Family Medicine, McMaster University, 100 Main Street West, Hamilton, Canada; 5 Department of Medicine, Division of Palliative Care, University of Ottawa, Ottawa, Canada; 6 Department of Health Sciences, Wilfrid Laurier University, 75 University Ave W, Waterloo, Canada; 7 School of Public Health Sciences, University of Waterloo, 200 University Ave W, Waterloo, Canada

**Keywords:** interRAI, Prognosis, End-of-life, Home care, Assessment

## Abstract

**Background:**

Prognostic accuracy is important throughout all stages of the illness trajectory as it has implications for the timing of important conversations and decisions around care. Physicians often tend to over-estimate prognosis and may under-recognize palliative care (PC) needs. It is therefore essential that all relevant stakeholders have as much information available to them as possible when estimating prognosis.

**Aims:**

The current study examined whether the interRAI Changes in Health, End-Stage Disease, Signs and Symptoms (CHESS) Scale is a good predictor of mortality in a known PC population and to see how it compares to the Palliative Performance Scale (PPS) in predicting 90-day mortality.

**Methods:**

This retrospective cohort study used data from 2011 to 2018 on 80,261 unique individuals receiving palliative home care and assessed with both the interRAI Palliative Care instrument and the PPS. Logistic regression models were used to evaluate the relationship between the main outcome, 90-day mortality and were then replicated for a secondary outcome examining the number of nursing visits. Comparison of survival time was examined using Kaplan-Meier survival curves.

**Results:**

The CHESS Scale was an acceptable predictor of 90-day mortality (c-statistic = 0.68; p < 0.0001) and was associated with the number of nursing days (c = 0.61; p < 0.0001) and had comparable performance to the PPS (c = 0.69; p < 0.0001). The CHESS Scale performed slightly better than the PPS in predicting 90-day mortality when combined with other interRAI PC items (c = 0.72; p < 0.0001).

**Conclusion:**

The interRAI CHESS Scale is an additional decision-support tool available to clinicians that can be used alongside the PPS when estimating prognosis. This additional information can assist with the development of care plans, discussions, and referrals to specialist PC teams.

## Background

The number of individuals with chronic progressive diseases such as cancer, organ failure, Alzheimer’s dementia, and chronic lung diseases is increasing worldwide. Each year, individuals with these types of chronic illnesses account for approximately 71% of all deaths[1, 2]. These individuals usually have long-term health issues before death and require specialized palliative care (PC) that adequately addresses their symptoms and needs[3].

Many serious illnesses tend to be quite complex, have a less predictable trajectory towards death and can therefore make it difficult to estimate prognosis[4, 5]. While there is debate about the relevance of prognosis to define the need for PC, it continues to play a role in clinical practice. In some countries, such as the US, prognosis is needed for determining hospice eligibility as individuals are only eligible for hospice if they have a prognosis of < 6 months to live[6]. A number of studies have found that clinicians tend to be overly optimistic in predicting survival times. A recent systematic review found that clinical accuracy of categorical estimates of survival ranged from 23% up to 78%, with prediction tending to be over-estimated by most clinicians[7].

There are tools to help improve prognostication, including the Palliative Performance Scale (PPS). The PPS, a modification of the Karnofsky Performance Scale is widely used in several countries (e.g., Canada, the US) as a tool to identify the needs for PC in a variety of health care settings (e.g., home care, long-term care, hospital, hospice) as the PPS has been found to be predictive of survival[8–10]. The PPS can be administered by nurses or physicians at any point during the illness trajectory and is based on five observable parameters, including the degree of ambulation, activity and extent of disease, ability to do self-care, food/fluid intake, and state of consciousness[11]. Since 2007 in Ontario, the PPS has been used for all outpatient cancer clinics or for individuals with cancer receiving care at home[12]. While some studies have shown the PPS to be a good predictor of morality in both cancer and non-cancer illnesses[8, 13–15], others have found it may have lower predictive validity for individuals with other life-limiting illnesses[16, 17].

In Ontario, the interRAI Palliative Care instrument (interRAI PC) is widely used among palliative home care clients[18]. interRAI is a not-for-profit network of over 140 clinicians, researchers, and policy experts from approximately 35 countries. interRAI’s research focuses on the development and application of standardized assessments to inform clinical, management, and policy decisions in populations with complex needs of all ages, including palliative care clients. The interRAI instruments have been shown to have strong reliability and validity, and they include a series of common items that are relevant in all health care settings[19–34]. The interRAI PC includes the Changes in Health, End-Stage Disease, Signs and Symptoms (CHESS) Scale, which was initially developed with individuals in complex continuing care (CCC) and was found to be a strong predictor of mortality in several studies[27, 29, 30, 35–40]. Its utility is not limited to cancer patients since it was shown to predict mortality in 11 neurological conditions in home care, long-term care and CCC settings[24] and it had better performance than the NYHA functional classification in heart failure[37]. However, its use in palliative home care has not been explored. Although the CHESS Scale has been found to be a valid predictor of mortality in a variety of populations in diverse settings, it is important to explore how well the scale works in predicting prognosis in a palliative home care population.

A recent study found that over a third of individuals receiving palliative home care in Ontario had a prognosis of more than six months to live, and while the majority had a cancer diagnosis (83%), both circulatory and musculoskeletal conditions were also highly prevalent[41]. Therefore, it is important to explore how well the CHESS Scale works in predicting prognosis in a heterogeneous palliative home care population. The main objective of the current study was to examine whether the CHESS Scale is a good predictor of mortality in a known palliative home care population and whether adding additional items and scales embedded within the interRAI PC assessment to the CHESS Scale could improve its ability to predict mortality. A secondary objective was to examine how the CHESS Scale compares with the PPS in predicting mortality and how it may be associated with days of nursing service use.

## Methods

### Study design

This retrospective cohort study utilized anonymized, secondary data collected using the interRAI PC assessment in Ontario. The interRAI PC is a comprehensive assessment instrument that identifies person-specific PC preferences, symptoms and needs to support clinicians in the care planning process[42]. The assessment includes roughly 250 items capturing key domains such as pain, physical symptoms, communication, prognosis, cognitive and physical functioning, mood, psychosocial well-being, and spirituality. Assessments are completed by trained care coordinators (e.g., registered nurses, social workers, and other allied health professionals) and are based on information from direct interviews and observations, available medical records, and discussions with the client, members of their health care team, and their informal care providers[43, 44]. Along with all of the information obtained from the person, their care providers, etc., care coordinators ultimately use their clinical judgement when determining a score for each item/scale. Assessors are not able to complete the assessment until all fields have been given a value, therefore missing data are virtually nonexistent. Once completed, data elements within the assessment automatically generate a series of health index scales (e.g., CHESS Scale, mood disturbance) that can be used to develop and evaluate care plans. Evidence of the validity and reliability of the interRAI PC instrument has been reported previously[43–46].

### Sample

All interRAI PC assessments completed between 2011 and 2018 were included, representing the most recent data available for Ontario. Data collected prior to 2011 was part of the pilot implementation of the interRAI PC assessment in Ontario and was not included. Each client was assessed with both the interRAI PC assessment and also the PPS, which were both being routinely collected as part of normal clinical practice during this time period. For individuals with multiple assessments within this time frame, the first assessment in the data for each unique individual was used for analysis. This resulted in a total sample of 80,261 unique individuals. The project was reviewed and approved by the Research Ethics Board at the University of Waterloo (REB #:30173).

### Measures

One of the six health index scales which is automatically generated upon completion of the assessment is the CHESS Scale. This scale includes 12 items used to measure health instability and identify those at risk of mortality, including a subjective rating of prognosis, change in capacity for decision making, change in activities of daily living (ADL) status, vomiting, peripheral edema, dyspnea, end-stage disease, weight loss, insufficient fluid intake, dehydration, decrease in food or fluid intake, and fluid output exceeds input. The scale ranges from zero (no health instability) to five (severe health instability) and has been shown to be a significant predictor of mortality. In post-acute settings, each one-point increase on the CHESS Scale is associated with a nearly two-fold increased risk of death[35].

The PPS ranges in 10% increments from 0 to 100%. A score of 0% indicates death, 10% indicates a totally bed-bound individual who is unable to do any activity and needs total assistance, and 100% indicates the person is able to carry on normal activities and to work without any special care. PPS scores are often stratified into three groups to identify changes in the individual’s condition, where scores of 70–100% are classified as stable, 40–60% as transitional and 10–30% as end-of-life[12, 47]. The PPS has been shown to be a predictor of mortality in a heterogeneous PC population, including care provided to individuals in the home, nursing home, hospital acute care setting and free-standing hospice units[8]. interRAI PC and PPS data were obtained from Ontario Health Shared Services (OHSS), the organization responsible for overseeing home care. The de-identified data were made available as part of an existing data sharing agreement between interRAI Canada and OHSS.

### Outcomes

The main outcome of interest of the current study was 90-day mortality. Byrne et al. proposed a ninety-day mortality as the preferred outcome indicator for local analysis and public reporting[48]. Ninety-day mortality refers to death occurring within 90 days based on administrative records maintained by OHSS. For all PC clients, the administrative database includes discharge date (if applicable) and discharge disposition, which is typically death in palliative clients, but can also include discharge to hospital, long-term care facility or residential hospice. Time to death was used in survival models with censoring at 90 days for those who lived beyond 90 days after the assessment. Persons discharged for reasons other than death before 90 days were censored at the discharge date.

A secondary outcome of interest examined the association of nursing service visits. Nursing services were chosen as an example of home care services that would be responsive to clinical complexity. In the general long-stay home care population, access to nurses is limited mainly to post-acute care clients (e.g., for surgical wound care, IV medication administration). However, palliative home care clients in Ontario are more likely to receive nursing services as their level of clinical complexity increases[49]. Nursing service use was based on the frequency of services provided at the time of the interRAI PC assessment. The nursing service utilization variable was based on the number of nursing visits that occurred in the last seven days prior to the assessment date. Care coordinators have access to administrative records as a source of information about the use of publicly-funded services and they also obtain information from the person and family members about other services that may have been paid for privately. The interRAI PC item includes both publicly-funded and private pay nursing services.

### Other measures

There are six other health index scales embedded within the interRAI PC assessment which are automatically generated upon completion of the assessment:

1. *The Cognitive Performance Scale (CPS)* is scored from zero to six and includes items on short-term memory, cognitive skills for daily decision making, independence in eating and expressive communication. The CPS has been validated against the Mini Mental State Exam (MMSE) and is correlated with the Montreal Cognitive Assessment (MoCA)[50].
2. *The Activities of Daily Living (ADL) Short Form* is scored from zero to 16 and includes items around personal hygiene, locomotion, toilet use and eating. Higher scores on the scale indicate a greater dependence on others to complete these ADLs[51].
3. *The Instrumental ADL (IADL) Capacity Scale* rates three IADLs (meal preparation, ordinary housework, and managing medications) on a scale from zero (independent) to six (total dependence on others). Higher scores on the scale represent greater difficulty in completing these tasks independently[23].
4. *The Pressure Ulcer Risk Scale (PURS)* is scored from zero to eight and includes items such as impaired bed mobility, weight loss, a history of unresolved pressure ulcers and bowel incontinence[52].
5. *The Pain Scale* is scored from zero (no pain/less than daily pain) to four (severe/daily pain) and has been validated against the Visual Analog Scale. The scale includes two items which capture both the frequency and intensity of pain[53].
6. *The Depression Rating Scale (DRS)* is a 14-point summative scale across seven items relating to mood and behavior. A score of three or higher has been found to be predictive of a clinical diagnosis of depression[54–58].

### Analysis

Descriptive analyses of categorical variables were used to provide an overview of the socio-demographic characteristics of the study population using the chi-square statistic. Due to the large sample size and potential for type I error, unadjusted odds ratios (OR) and 95% confidence intervals (CI) were also generated to help identify statistically important differences between individuals who did and did not die within 90-days. Candidate independent variables that could be used in addition to the CHESS Scale were identified by examining the relationship between the PPS and interRAI PC items as well as those that have been identified in the existing literature as being potentially important. In addition to the CHESS Scale, a number of other interRAI PC items that were explored in relation to the PPS included age, sex, the other health index scales (ADL Short Form, IADL Capacity Scale, CPS, DRS, Pain Scale and PURS), fluctuating consciousness, fatigue, mode of nutritional intake and physical improvement potential (from the perspective of both the person and the caregiver). A Pearson correlation coefficient of 0.2 or greater was used to determine a small effect size. Since we were interested in exploring all meaningful correlations between interRAI PC items and the PPS, we chose a more conservative effect size of 0.2[59]. All of the candidate independent variables met this criterion except for the DRS and Pain Scale, which were therefore dropped from further analyses (Table 1).

**Table 1:** Pearson correlation coefficients of interRAI PC items in relation to the PPS

interRAI PC item/scale	Pearson correlation coefficients	p-value
Activities of Daily Living (ADL) Short Form	−0.73	< 0.0001
Instrumental Activities of Daily Living (IADL) Capacity Scale	−0.65	< 0.0001
Pressure Ulcer Risk Scale (PURS)	−0.58	< 0.0001
Changes in Health, End-Stage Disease, Signs and Symptoms (CHESS) Scale	−0.51	< 0.0001
Cognitive Performance Scale (CPS)	−0.42	< 0.0001
Fatigue	−0.40	< 0.0001
Nutritional intake (requires modification)	−0.36	< 0.0001
Fluctuating conscious	−0.32	< 0.0001
Physical improvement potential according to the caregiver	0.23	< 0.0001
Physical improvement potential according to the patient	0.22	< 0.0001
Age	−0.21	< 0.0001
Depression Rating Scale (DRS)	−0.11	< 0.0001
Pain scale	−0.10	< 0.0001
Sex	−0.02	< 0.0001

Mortality was treated as a binary variable with death within 90 days (yes/no) as the main dependent variable of interest. Logistic regression models were used to estimate odds ratios and obtain c statistics. The c statistic represents the area under the curve when plotting a receiver operating characteristic curve. It provides an estimate of the model’s ability to discriminate between outcomes (e.g., died yes/no). In addition, survival plots with Kaplan-Meier curves were used to compare mortality patterns for the PPS and CHESS Scale.

The modelling for mortality focused on a few comparisons of interest, including: (a) Model 1: the performance of the full versions of the PPS (0-100%); (b) tests of alternative cut-points for the PPS that have been proposed in the literature[12, 47] (e.g., Model 2: 70-100% vs. 10-39% vs. 40-69%; and Model 3: 70-100% vs. 10-49% vs. 50-69%; c) Model 4: CHESS Scale (0–5) on its own; and d) Model 5: model that includes the CHESS Scale and other interRAI PC covariates (listed above) associated with mortality. The odds ratios for Models 1 through 4 are unadjusted as we were interested in seeing how the various cut-points of the PPS and CHESS Scale influenced the outcome on their own. Only Model 5 shows adjusted odds ratios as it is adjusted for all variables included in the model. Although the odds ratios associated with the covariates examined provide information about the direction and magnitude of associations, the c statistic is of particular interest as a means of direct comparison of the relative performance of the PPS, CHESS Scale and a fuller model with additional covariates.

The final multivariable models were developed by examining bivariate odds ratios of potential covariates and then reduction of multivariate models to include only those that were significant at the 0.05 level. Models were reduced manually (rather than with automated stepwise procedures) and alternate models were tested to ensure order of entry/deletion effects did not occur. For variables that had potential problems with collinearity (e.g., ADL and pressure ulcers), alternative models were tested for each variable in question and the model with the best ability to discriminate based on the c-statistic[60] was retained if both variables were not significant.

We also examined nursing service use as a secondary outcome, which was defined as receipt of nursing visits on at least 3 of the last 7 days (yes/no). Our aim was to examine an additional proxy indicator of medical complexity with the assumption that factors associated with increased mortality risk would also drive increased use of nursing services. The five models listed above were replicated using receipt of nursing visits as the dependent variable to provide evidence of convergent validity that complements the evidence of predictive validity demonstrated in the relationship with mortality. All statistical analyses were done using SAS, version 9.4 (SAS Institute’s Inc.)[61]. The study followed the STrengthening the Reporting of OBservational studies in Epidemiology (STROBE) guidelines[62].

## Results

Table 2 provides an overview of bivariate associations with mortality. PC clients aged 85 years and older were more likely to die within 90 days compared to their younger counterparts (OR = 1.79; 95% CI: 1.63, 1.97). Clients that lived with their spouse/partner or lived with other relatives were more likely to die within 90 days compared to those living alone (OR = 1.33; CI: 1.27, 1.38 and OR = 1.38; CI: 1.32, 1.44). Females were less likely to die within this time frame (OR = 0.80; CI: 0.78, 0.83). Compared to those receiving in-patient hospice, individuals who were enrolled in any other type of palliative program (e.g., PC unit/bed, home hospice, outpatient PC) were all less likely to experience mortality within the last 90 days. With each increasing score on the CHESS Scale, the odds of dying within 90 days also increased. For example, compared to individuals with a CHESS score of 0, those with a score of 4 had a significantly increased odds of dying (OR = 11.54; CI: 10.03, 13.27), which further increased for those with a CHESS Scale score of 5 (OR = 18.05; CI: 15.64, 20.84). Similarly, individuals with lower PPS scores (i.e., worse functioning) were also significantly more likely to die compared to those with higher PPS scores (OR: 20.55; CI: 9.13, 46.23; Table 2).

**Table 2:** Characteristics of the study population (N = 80,261), stratified by mortality within 90 days (n = 29,682)

Item	Total sample (n = 80,261)	Died within 90 days (n = 29,682)	Odds ratio (95% CI)	p value
	**% (n)**			
**Age (years)**				
18–44	2.9 (2344)	28.4 (665)	Reference	Reference
45–64	25.3 (20,296)	33.7 (6838)	1.28 (1.17, 1.41)	< 0.0001
65–74	25.9 (20,807)	36.4 (7569)	1.44 (1.31, 1.59)	< 0.0001
75–84	27.4 (22,008)	38.5 (8467)	1.58 (1.44, 1.73)	< 0.0001
85+	18.4 (14,803)	41.5 (6142)	1.79 (1.63, 1.97)	< 0.0001
**Sex**				
Male	49.7 (39,891)	39.5 (15,769)	Reference	Reference
Female	50.3 (40,370)	34.5 (13,913)	0.80 (0.78, 0.83)	< 0.0001
**Living arrangement**				
Alone	19.1 (15,306)	31.6 (4839)	Reference	Reference
With spouse/partner	58.1 (46,599)	38.0 (17,701)	1.33 (1.27, 1.38)	< 0.0001
With other relatives	22.9 (18,356)	38.9 (7142)	1.38 (1.32, 1.44)	< 0.0001
**Type of palliative program**				
In-patient hospice	1.1 (915)	45.4 (415)	Reference	Reference
Palliative care unit or bed	1.3 (1050)	36.8 (386)	0.70 (0.59, 0.84)	0.0001
Home hospice	94.1 (75,528)	36.8 (27,770)	0.70 (0.62, 0.80)	< 0.0001
Outpatient palliative care	3.0 (2392)	41.5 (992)	0.85 (0.73, 1.00)	0.0434
Other	0.5 (376)	31.7 (119)	0.56 (0.43, 0.72)	< 0.0001
**Time since last hospital stay**				
No hospitalization within 90 days	44.0 (35,332)	34.0 (12,000)	Reference	< 0.0001
31 to 90 days ago	12.8 (10,267)	32.9 (3373)	0.95 (0.91, 1.00)	0.0361
15 to 30 days ago	12.0 (9594)	35.6 (3418)	1.08 (1.03, 1.13)	0.0024
8 to 14 days ago	12.3 (9857)	40.6 (3998)	1.33 (1.27, 1.39)	< 0.0001
In the last 7 days	15.8 (12,683)	46.4 (5887)	1.68 (1.62, 1.76)	< 0.0001
Now in hospital	3.2 (2528)	39.8 (1006)	1.29 (1.18, 1.40)	< 0.0001
**Disease diagnosis**				
Non-cancer	15.5 (12,475)	37.0 (4613)	Reference	Reference
Cancer	84.5 (67,786)	37.0 (25,069)	1.00 (0.96, 1.04)	1.0000
**Changes in Health, and End-Stage Disease, Signs and Symptoms (CHESS) Scale**				
0 (stable)	4.0 (3006)	7.3 (220)	Reference	Reference
1	10.4 (7872)	13.9 (1093)	2.04 (1.78, 2.38)	< 0.0001
2	18.4 (13,962)	22.0 (3077)	3.58 (3.10, 4.13)	< 0.0001
3	26.6 (20,195)	33.3 (6733)	6.33 (5.50, 7.29)	< 0.0001
4	28.6 (21,763)	47.7 (10,376)	11.54 (10.03, 13.27)	< 0.0001
5 (highly unstable)	12.2 (9237)	58.9 (5429)	18.05 (15.64, 20.84)	< 0.0001
**Palliative Performance Scale (PPS)**				
100% (high functional status)	0.1 (63)	11.1 (7)	Reference	Reference
90%	0.7 (438)	4.6 (20)	0.38 (0.16, 0.95)	0.0378
80%	3.9 (2486)	10.5 (262)	0.94 (0.43, 2.09)	0.8840
70%	13.2 (8492)	16.7 (1418)	1.60 (0.73, 3.53)	0.2400
60%	23.9 (15,421)	25.5 (3931)	2.74 (1.25, 6.01)	< 0.0001
50%	30.7 (19,759)	39.6 (7816)	5.23 (2.39, 11.49)	< 0.0001
40%	17.4 (11,221)	54.5 (6116)	9.58 (4.37, 21.05)	< 0.0001
30%	7.4 (4745)	60.7 (2878)	12.33 (5.61, 27.12)	< 0.0001
20%	2.1 (1358)	74.9 (107)	23.86 (10.77, 52.85)	< 0.0001
10% (low functional status)	0.7 (471)	72.0 (339)	20.55 (9.13, 46.23)	< 0.0001

### Comparing PPS and interRAI PC models to predict mortality

Table 3 shows logistic regression models based on the PPS and the interRAI PC to predict 90-day mortality. The full version of the PPS (Model 1) was found to be an acceptable predictor of mortality (c-stat = 0.69; p < 0.0001). When the PPS score was stratified into known cut-points suggested in the literature, the two models performed slightly worse than the full version of the PPS. For example, in Model 2 (70-100%, 10-39% and 40-69%), the model’s predictive ability was slightly less than the non-stratified PPS (Model 1; c-stat = 0.63 vs. 0.69). Similar results were also observed for Model 3 (70-100%, 10-49% and 50-69%; c-stat = 0.66 vs. c-stat of full PPS = 0.69). Model 4, performed similarly to the full PPS model (c-stat = 0.68 vs. 0.69). Finally, when the CHESS Scale was combined with additional interRAI PC items (e.g., age, ADL, IADL, nutritional intake, fluctuating consciences, fatigue, pain, and physical improvement potential; Model 5), the model was better at predicting 90-day mortality (c-stat = 0.72; p < 0.0001) compared to all PPS models (Models 1–3) and the model with CHESS on its own (Model 4). Within this model, the strongest predictors of survival were the CHESS score, age, and mode of nutritional intake (Table 3).

**Table 3:** Logistic regression analysis assessing models to predict mortality within 90 days in palliative home care clients

MODEL 1: Full PPS (reference = 10)^a^		MODEL 2: PPS collapsed (reference = 70–100)		MODEL 3: PPS collapsed (reference = 70–100)		MODEL 4: interRAI CHESS (reference = 0)		MODEL 5: CHESS & additional interRAI PC items	
Variable	Unadjusted OR (95% CI)	Variable	Unadjusted OR (95% CI)	Variable	Unadjusted OR (95% CI)	Variable	Unadjusted OR (95% CI)	Variable	Adjusted OR (95% CI)
100**	0.05 (0.02, 0.11)	40–69	3.36 (3.39, 3.78)	50–69	2.87 (2.71, 3.04)	1	2.04 (1.76, 2.38)	CHESS 1	1.73 (1.48, 2.02)
90	0.02 (0.01, 0.03)	10–39	10.4 (9.64, 11.13)	10–49	7.96 (7.50, 8.44)	2	3.58 (3.10, 4.13)	CHESS 2	2.50 (2.16, 2.90)
80	0.05 (0.04, 0.06)					3	6.33 (5.50, 7.29)	CHESS 3	3.74 (3.24, 4.32)
70	0.08 (0.06, 0.10)					4	11.5 (10.0, 13.3)	CHESS 4	5.54 (4.80, 6.40)
60	0.13 (0.11, 0.16)					5	18.1 (15.6, 20.8)	CHESS 5	6.49 (5.59, 7.54)
50	0.26 (0.21, 0.31)							Sex	0.84 (0.81, 0.87)
40	0.47 (0.38, 0.57)							Age (45–64)	0.96 (0.86, 1.07)
30	0.60 (0.49, 0.74)							Age (65–74)	1.12 (1.00, 1.23)
20	1.16 (0.92, 1.47)							Age (75–84)	1.18 (1.06, 1.31)
								Age (85+)	1.18 (1.06, 1.31)
								ADL	1.03 (1.02, 1.03)
								IADL	1.06 (1.05, 1.06)
								Nutritional intake 1	1.16 (1.11, 1.20)
								Nutritional intake 2	2.60 (1.99, 3.39)
								Fluctuating consciousness*	1.10 (1.02, 1.17)
								Fatigue	1.12 (1.10, 1.14)
								Pain	1.08 (1.07, 1.10)
								Physical improvement potential (person)	0.77 (0.74, 0.80)
								Physical improvement potential (caregiver)	0.71 (0.67, 0.75)
**X** ^**2**^	< 0.0001		< 0.0001		< 0.0001		< 0.0001		< 0.0001
** C-stat**	0.692		0.625		0.664		0.683		0.723

^a^ Note that different reference groups are used for models 1–4 due to sample size limitations in the highest PPS scores.

*p < 0.05; **p < 0.001; All other variables had a p < 0.0001, except age [45–64], which was not significant.

Abbreviations: interRAI PC = interRAI Palliative Care instrument; OR = Odds ratio; CI = Confidence interval; X^2^: Chi Square.

**Fig. 1:**
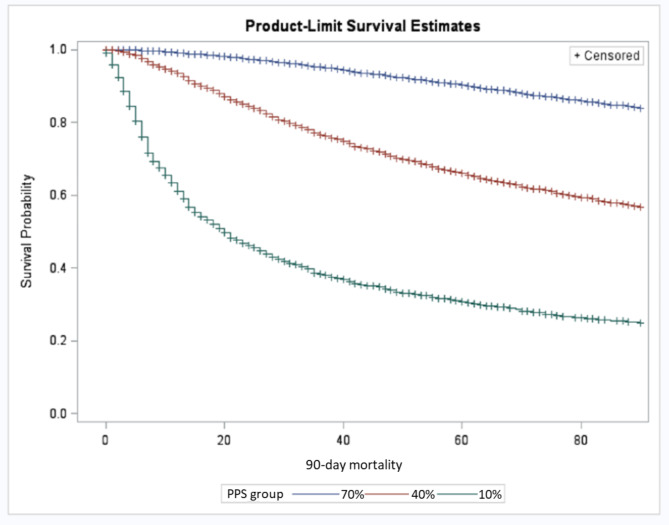
Survival estimates among palliative home care clients in Ontario based on the Palliative Performance Scale (PPS)

**Fig. 2:**
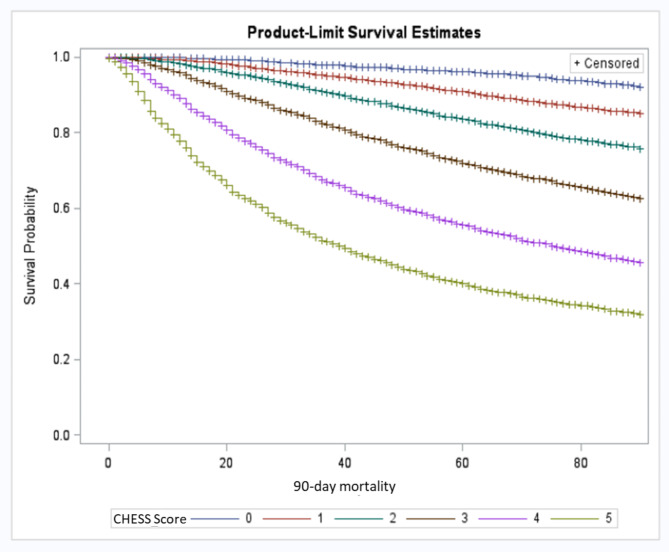
Survival estimates among palliative home care clients in Ontario based on the interRAI CHESS Scale

The Kaplan-Meier survival curves stratified by the collapsed version of the PPS (40% and 70% cut-offs) and the CHESS Scale are shown in Figs. 1 and 2, respectively. Individuals with a PPS score of 70–100% had a 90-day survival probability of 84%, while the probability for those with a PPS score of 40-69% was 59%, which dropped to 31% for individuals in the lowest PPS cut-off (long-rank test < 0.0001; Fig. 1). The probability of survival to 90 days for a CHESS Scale score of 0 was 92%, which dropped to 65% for those with a score of 3 and went down to 38% for individuals with a CHESS Scale score of 5. For every one-point increase on the CHESS Scale, there was a nearly two-fold increased risk of mortality within 90-days (log-rank test < 0.0001; Fig. 2).

### Comparing PPS and interRAI PC models and its association with the number of nursing days

Table 4 presents logistic regression models based on the PPS and the interRAI PC to examine the association with the number of nursing days in palliative home care clients. The five models that were used to predict mortality were replicated to see if they were also associated with the number of nursing days. The full PPS model (Model 1) had the strongest association when examining the number of nursing days compared to all other models (c-stat = 0.69, p < 0.0001)^2^. The collapsed versions of the PPS (Models 2 and 3) performed worse than the full model (PPS cut-points 10% and 40%: c-stat = 0.61; p < 0.0001; PPS cut-points 10% and 50%: c-stat = 0.64; p < 0.0001). Model 4 which used the CHESS Scale had the lowest c-statistic of all models (c-stat = 0.61; p < 0.0001), while the model combining the CHESS Scale with other interRAI PC items performed better than both the collapsed versions of the PPS (c-stat = 0.68; p < 0.0001; Table 4).

**Table 4:** Logistic regression analysis assessing models associated with the receipt of nursing visits in palliative home care clients

MODEL 1: PPS (reference = 10)^a^		MODEL 2: PPS collapsed (reference = 70–100)		MODEL 3: PPS collapsed (reference = 70–100)		MODEL 4: interRAI CHESS (reference = 0)		MODEL 5: CHESS & additional interRAI PC items	
Variable	Unadjusted OR (95% CI)	Variable	Unadjusted OR (95% CI)	Variable	Unadjusted OR (95% CI)	Variable	Unadjusted OR (95% CI)	Variable	Adjusted OR (95% CI)
100	0.06 (0.02, 0.14)	40–69	1.42 (1.33, 1.51)	50–69	1.98 (1.87, 2.10)	1	1.51 (1.34, 1.70)	CHESS 1	1.31 (1.16, 1.48)
90	0.09 (0.06, 0.13)	10–39	1.79 (1.62, 1.51)	10–49	5.26 (4.96, 5.59)	2	2.01 (1.80, 2.26)	CHESS 2	1.51 (1.34, 1.70)
80	0.09 (0.07, 0.11)					3	2.61 (2.34, 2.92)	CHESS 3	1.73 (1.54, 1.94)
70	0.12 (0.10, 0.15)					4	3.62 (3.25, 4.05)	CHESS 4	2.04 (1.81, 2.29)
60	0.18 (0.15, 0.21)					5	5.06 (4.52, 5.68)	CHESS 5	2.07 (1.83, 2.34)
50	0.27 (0.23, 0.33)							Sex	1.03 (0.99, 1.06)
40	0.49 (0.41, 0.59)							Age (45–64)	0.89 (0.81, 0.98)
30	0.76 (0.63, 0.92)							Age (65–74)	0.67 (0.61, 0.74)
20	1.18 (0.95, 1.47)							Age (75–84)	0.77 (0.70, 0.85)
								Age (85+)	0.62 (0.56, 0.68)
								ADL	1.06 (1.05, 1.06)
								IADL	1.04 (1.04, 1.04)
								Nutritional intake 1	2.05 (1.63, 2.57)
								Nutritional intake 2	1.18 (1.13, 1.22)
								Fluctuating consciousness*	1.05 (0.98, 1.12)
								Fatigue	1.06 (1.05, 1.08)
								Pain	1.13 (1.12, 1.15)
								Physical improvement potential (person)	1.12 (1.07, 1.17)
								Physical improvement potential (caregiver)	1.18 (1.11, 1.25)
**X** ^**2**^	< 0.0001		< 0.0001		< 0.0001		< 0.0001		< 0.0001
** C-stat**	0.692		0.610		0.644		0.607		0.668

^a^ Note that different reference groups are used for models 1–4 due to sample size limitations in the highest PPS scores.

*p < 0.05; **p < 0.001; All other variables had a p < 0.0001, except for all levels of age, which was not significant.

Abbreviations: interRAI PC = interRAI Palliative Care instrument; OR = Odds ratio; CI = Confidence interval; X^2^: Chi Square.

## Discussion

The interRAI CHESS Scale has been found to be a very good predictor of mortality in a variety of settings, including complex continuing care, home care and long-term care. In the current study, we found that the CHESS Scale is an acceptable predictor of mortality in a heterogeneous palliative home care population. With each one-point increase on the CHESS Scale, there was a nearly two-fold increased risk of mortality within 90-days. Additionally, the results of the current study show that the CHESS Scale is comparable to the PPS in predicting 90-day mortality. This was especially true when the CHESS Scale was combined with other items from the interRAI PC, including age, sex, ADLs, IADLs, nutritional intake, fluctuating consciousness, fatigue, pain, and physical improvement potential. When combined, this model performed slightly better, in terms of discrimination, than the PPS.

The interRAI PC CHESS Scale and other assessment items were also associated with clinical resource use and are therefore informative for analyses related to costs of palliative home care[49]. Performance was comparable to the un-collapsed version of the PPS and notably better than the collapsed alternatives.

Most prognostic tools have been developed to identify individuals who are imminently or actively dying, and have typically focused on those with a cancer diagnosis[63, 64]. The “surprise question” (“Would you be surprised if the person died in the next 12 months”)[65] has often been used to identify individuals eligible for PC services, although recent data have shown that it is not particularly accurate at identifying individuals near the end-of-life[66]. There are a number of other prognostic tools that have been developed for use in both the hospital and community setting[67, 68]. However, the CHESS Scale is more advantageous as it has been shown to predict mortality in a number of heterogeneous populations, including those in home care, long-term care and CCC settings. A recent study examining the trajectory of end-of-life pain and other physical symptoms in individuals with cancer found that the CHESS Scale continually increased during the last two months of life and identified 50% of the cohort with high health instability in the last weeks of life[69]. The CHESS Scale has also been used to predict mortality in individuals with other non-cancer diagnoses, which is beneficial over other disease-specific tools, as this allows for direct comparisons across various diagnostic groups[24].

The CHESS Scale is a decision support tool that is available to clinicians when they complete a number of interRAI instruments (e.g., home care, long-term care), to inform shared decision-making. The interRAI Home Care (HC) assessment is currently being used in 20 other countries besides Canada. This allows for HC clinicians to gain some insight into the mortality risk for individuals with a life-limiting illness, even if they have not been identified as eligible for palliative care. Additionally, given its ability to predict mortality, the CHESS Scale can help clinicians decide when to initiate discussions around advance care planning with the individual and their family. For regions that do not currently use the PPS on a regular basis, but are routinely using an interRAI assessment, the CHESS Scale can be calculated and provide information about mortality risk similar to the PPS. The interRAI suite of assessments are used widely in Canada and internationally, therefore the CHESS Scale represents an efficient way to predict mortality without administering additional assessments, thereby potentially reducing assessment burden for individuals and their families.

Home-based palliative nursing care at the end-of-life typically focuses on alleviating distressing symptoms for the individual and their family and ensuring optimal quality of life. When these services are delivered early in the illness trajectory, it has been shown to reduce late-life health service use (i.e., hospital admissions)[63]. We found that the PPS, CHESS Scale, and other interRAI PC items were associated with increased odds of use of home nursing services, which are infrequently provided to other home care clients in Canada[39]. This suggests that the interRAI PC could also be used to predict clinical resource use[49] and it could service as a decision support tool to identify persons with complex needs who may be under-served with nursing services.

Prognosis has typically been used as the benchmark for when to initiate PC and PC services, and while it is important, it should not be the sole determinant of when to initiate PC. The care needs of both the individual and their family should also be considered when discussing the initiation of PC. PC can begin at the time of diagnosis and can be provided in conjunction with curative treatments. Having access to PC services early in the course of the illness can facilitate care planning, which helps ensure that care is in line with the preferences of both the individual and their family[70, 71]. Additionally, early PC can enable appropriate referrals to specialist PC services, which have been shown to improve mood, assist with advance care planning, reduce health care utilization and improve overall quality of life[72].

An additional use of the CHESS Scale and other interRAI measures is for the specification of reassessment intervals in palliative care. In order to track clinical outcomes and to adjust care plans in response to changing health needs, reassessments should occur on a timely basis. In long-stay home care clients, the recommended reassessment interval is six months. However, this would be too long for most palliative clients in the community. CHESS scores and other interRAI PC indicators could be used proactively to schedule the reassessment cycle to ensure that the needs of palliative clients are met on an ongoing basis.

Future research with these data will focus on the development of palliative-specific variants of the CHESS Scale that employ more items from the interRAI PC into a single composite measure of mortality risk and clinical complexity. In addition, we will explore the use of the CHESS Scale and other interRAI PC measures to develop palliative specific case-mix classification systems that can be used for resource allocation.

There are several strengths in the current study, including the large sample size, which represents most regions in Ontario as the majority are using the interRAI PC assessment for individuals receiving palliative home care. Additionally, while this paper focuses on PC data, the CHESS Scale can also be calculated using other interRAI instruments that are in widespread use internationally. A potential limitation is that the data were limited to individuals receiving PC in Ontario only, as no other provinces or territories in Canada are currently using the interRAI PC assessment. However, other countries such as New Zealand have implemented the interRAI PC assessment nationally, which will allow for cross-national comparisons to be made in the future. Finally, we chose to utilize the first available assessment within the data for each unique individual, which may not have captured the most current situation for each person. We felt it was important to include the first assessment for everyone as this likely represents the beginning of the receipt of palliative home care services for these individuals.

## Conclusion

The interRAI CHESS Scale was an acceptable predictor of mortality in a palliative home care population with comparable performance to the PPS. The CHESS Scale is available on most interRAI instruments, which are widely used across Canada and globally. Having access to this important-decision support tool in multiple sectors of the health care system (e.g., home care, long-term care, etc.), allows for the potential for individuals with serious and life-limiting illnesses to be identified earlier. This provides clinicians with upstream information to assist with care planning, timing of when to initiate PC and conversations around advance care planning. Having these conversations early on and throughout the illness trajectory can optimize the quality of care being provided to the individual and their family.

## Data Availability

The data are not publicly available, but they are directly available to interRAI fellows and their staff and students. Other researchers can access the data from the Canadian Institute for Health Information for researchers who meet the criteria for access to confidential data. These data represent third party data that are not owned nor collected by the study authors. A data request form can be found here: https://www.cihi.ca/en/access-data-and-reports/make-a-data-request.

## Footnotes

2 Note that model 1 uses PPS = 10 as the reference group due to cell size limitations for PPS = 100. This results in inverse Odds Ratios to those shown in Models 2 to 4.
